# Predictors of medication adherence among patients with severe psychiatric disorders: findings from the baseline assessment of a randomized controlled trial (Tecla)

**DOI:** 10.1186/s12888-018-1737-4

**Published:** 2018-05-29

**Authors:** Ulrike Stentzel, Neeltje van den Berg, Lara N. Schulze, Thea Schwaneberg, Franziska Radicke, Jens M. Langosch, Harald J. Freyberger, Wolfgang Hoffmann, Hans-Jörgen Grabe

**Affiliations:** 1 Institute for Community Medicine, University Medicine Greifswald, Ellernholzstraße 1-2, 17487 Greifswald, Germany; 2 Department of Psychiatry and Psychotherapy, University Medicine Greifswald, Ellernholzstraße 1-2, 17487 Greifswald, Germany; 3 Department of Vascular Medicine, University Heart Center Hamburg, University Medical Center Hamburg-Eppendorf (UKE), Martinistraße 52, 20246 Hamburg, Germany; 4 Bethanien Hospital for Psychiatry, Psychosomatics and Psychotherapy, Gützkower Landstraße 69, 17489 Greifswald, Germany; 5 HELIOS Hanseklinikum Stralsund, department for psychiatry and psychotherapy, Rostocker Chaussee 70, 18437 Stralsund, Germany

**Keywords:** Psychiatry, Mental health disorders, Schizophrenia, Psychotic disorders, Bipolar disorders, Adherence, Non-adherence, MARS-D

## Abstract

**Background:**

Schizophrenia and bipolar disorder are characterized by a high disease burden. Antipsychotic medication is an essential part of the treatment. However, non-adherence is a major problem. Our aim was to examine potential determinants of non-adherence for patients with severe mental disorders.

**Methods:**

Baseline data of the study “Post stationary telemedical care of patients with severe psychiatric disorders” (Tecla) were used. Medication adherence was assessed with the Medication Adherence Report Scale German version (MARS-D). A logistic regression was calculated with age, sex, education, employment status, level of global functioning, social support and intake of typical and atypical antipsychotics as predictors.

**Results:**

*N* = 127 participants were included in the analysis (*n* = 73 men, mean age 42 years). The mean MARS-D Score was 23.4 (SD 2.5). The most common reason for non-adherence was forgetting to take the medicine. Significant positive determinants for adherence were older age (OR 1.02, 95% CI 1.011–1.024, *p* < 0.0001), being employed (OR 2.46, 95% CI 1.893–3.206, *p* < 0.0001), higher level of global functioning (overall measure of how patients are doing) (OR 1.02, 95% CI 1.012–1.028, *p* < 0.0001), having social support (OR 1.02, 95% CI 1.013–1.026, *p* < 0.0001), and intake of typical antipsychotics (OR 2.389, 95% CI 1.796–3.178, *p* < 0.0001). A negative determinant was (female) sex (OR 0.73, 95% CI 0.625–0.859, *p* = 0.0001).

**Conclusions:**

Especially employment, functioning and social support could be promising targets to facilitate adherence in patients with schizophrenia or bipolar disorder.

**Trial registration:**

This study is retrospectively registered at the German Clinical Trials Register with the trial registration number DRKS00008548 at 21/05/2015.

## Background

Schizophrenia as well as other psychotic disorders and bipolar disorders are serious mental diseases. In Germany, 19 new schizophrenia-cases per 100,000 people per year are diagnosed. In Germany, the 12-month-prevalence of schizophrenia and other psychotic disorders is estimated 2.6% and of bipolar disorders 1.5% [1]. The disease burden is high for mental disorders. The number of days with limitations is 3 times higher for people with mental disorders compared to healthy persons [1] and schizophrenia is one of the ten diseases with the highest number of years of life lived with disability (YLD) [2].

Medication is an essential part of the treatment of schizophrenia and bipolar disorders; both in acute episodes and in long-term management. Several studies showed that the relapse rate is significantly lower with drug therapy [2–5], provided that the patient is adherent. Adherence is defined by the WHO as “the extent to which a person’s behavior – taking medication, following a diet, and/or executing lifestyle changes – corresponds with agreed recommendations from a health care provider” [6]. Non-adherent behavior increases the risk of relapses and rehospitalization [7–9]. However, non-adherence is one of the major problems in patients with schizophrenia and bipolar disorders [10]. The prevalence of non-adherence to antipsychotics ranges from 20 to 89% for patients with schizophrenia or bipolar disorders [11–13]. Dolder et al. examined adherence in an outpatient setting using prescription fill rates. Their results showed an adherence of 55% after 12 months among patients taking atypical antipsychotics (second generation) [14].

To reduce non-adherence in patients with severe mental disorders, it is necessary to know more about the reasons for non-adherence und to determine factors that influence adherence positively or negatively. A few studies have addressed specific factors determining adherence of patients with schizophrenia or with bipolar disorders [15–20]. However, the results of these studies often differ [16, 19, 20] and non-adherence is considered a multi-causal phenomenon [16].

The aim of this analysis is to identify possible determinants for non-adherence of patients with schizophrenia, other psychotic disorders and bipolar disorders, including age, sex, education, the status of employment, the level of functioning, presence of social support, adverse drug effects.

## Methods

### Patient sample and data

The data for this analysis were taken from the baseline assessment from an intervention study to evaluate telemedical care for patients with severe psychiatric disorders (“Post stationary telemedical care of patients with severe psychiatric disorders” (Tecla)). The goal of this project is to improve medication adherence for patients with severe psychiatric disorders on the basis of regular, individualized telephone calls and short-text-messages. Tecla is designed as a prospective controlled randomized intervention study. All participants receive computer assisted baseline and follow-up interviews after 3 and 6 months. The participants are recruited from three psychiatric departments in Western-Pomerania in the very northeast of Germany. Participants were patients in day-care hospitals or in open or closed inpatient wards. The recruitment occurs shortly before discharge and is done by a study psychologist. Inclusion criteria are a medical diagnosis of any form of schizophrenia (ICD-10 F20), schizoaffective disorders (ICD-10 F25), or bipolar disorders (ICD-10 F31), and age ≥ 18 years. Exclusion criteria were scheduled inpatient treatments within the next 6 months and missing accessibility by telephone. A comprehensive description of the study protocol for the Tecla study is published elsewhere [21].

Additionally, data from participants of the IMeS study (Approaches of individualized medicine in psychiatric disorders) were included. The aim of this study is to identify biomarkers from genetic material, and to examine metabolic processes and bodily protein in blood samples. The recruitment of the patients, the inclusion criteria and the baseline assessment of the IMeS-study are identical with the Tecla study. Both samples were collected at the same recruitment sites.

All participating patients gave their written informed consent. The data assessment and documentation were conducted based on eCRFs and an IT-supported documentation system [22].

### Measures

Medication adherence was measured with the Medication Adherence Report Scale, German version (MARS-D) that detects non-adherent behavior by self-report [23]. It is a measure for non-adherence in general, not for mental disorders in particular. The scale considers also the frequency of non-adherent behavior. The questions are formulated in a non-threatening and non-judgmental way to minimize social desirability bias [24, 25]. The original Medication Adherence Report Scale (MARS-5, in English language) was developed because patients tend to overestimate their adherence [26–28] or to conceal non-adherent behavior [23]. The MARS-D has 5 items to assess how the drugs were taken. The 5 items are “I forget to take my medication”, “I change the dose of my medication”, “From time to time I stop taking my medication for a while”, “I sometimes decide to skip the medication” and “I take less medication than I am instructed to.” The questions provide 5 answer categories from “always” to “never” (scored 1 to 5) so that the total score is between 5 (no adherence) and 25 (complete adherence) [29].

The Global Assessment of Functioning (GAF) is an overall measure of how patients are doing from positive mental health up to severe psychopathology [30]. It is known, that functioning is low in people with current mental health disorders, so functioning can be used as an expression of the severity of illness [31]. The GAF-questionnaire measures the degree of mental illness by rating psychological, social and occupational functioning [30] on an ordinal scale from 1 to 100 [32]. The scale is divided into 10-point intervals. The lowest interval (score 1 to 10) represents severe illness, the highest interval (score 91 to 100) represents the healthiest condition [30].

Social support was assessed using the measure F-SozU (Social support, short form with 14 items) [33]. The authors defined social support as the result of cognitive-emotional processing and assessment of current and past social interactions. The concept is based on cognitive approaches and assesses the subjective conviction to get support from the subject’s social network if necessary. This 14-item short form is appropriate for the assessment of a more generally perceived social support [33]. The statements refer to the fields of emotional support (to be liked and accepted by others, to share feelings, to experience participation), to provide practical assistance (practical help in everyday problems, for example to borrow things, getting practical advice, getting help with challenging tasks) and social integration (belonging to a circle of friends, doing joint ventures, knowing people with similar interests) and are assessed using a 5 category Likert-scale from “does not apply” (scored 1) to “applies exactly” (scored 5) [33].

Adverse drug effects were assessed using a 5 category Likert-scale including “no side effects”, “little”, “moderate”, “strong” and “very strong” for each of the following side effects: movement disorders, muscle stiffness, involuntary shiver, motionlessness, muscle spasm, agonizing restlessness/problems to sit still (can’t be suppressed at will), lack of sexual desire/loss of libido, increase in weight, increased appetite, heavy feeling of illness/chills/fever and milk flow [34].

To adjust for social desirability (defined as the “tendency to give overly positive self-descriptions” [35]), the Short Scale Social Desirability-Gamma (KSE-G) was used [36]. The KSE-G measures two aspects of social desirability: the exaggeration of positive qualities (PQ +), and the minimization of negative qualities (NQ-) [35]. Both aspects were assessed with three items each on a 5 category Likert-scale. The categories range from “does not apply” (score 0) to “fully applies” (score 4) [36].

The baseline assessment contained also a sociodemographic part to assess sex, age, education, and employment status. Patients’ diagnoses were extracted from medical files.

### Statistical analysis

To investigate determinants for medication adherence, a multivariate logistic regression approach was used. The MARS-D score was dichotomized in “adherent” and “non-adherent”. Following recent literature, the cut-off was set at a MARS-D score of 24. Participants with a MARS-D score of 25 were seen as adherent (coded as 1), participants with a score < 25 were considered as non-adherent (coded as 0) [37, 38]. A multiple imputation (based on the EM algorithm [39]) was performed to deal with missing data. Fifty-nine percent of the records where complete. Thirty percent of the records missed one and 11% of the records missed two or more items. There were no missing items regarding the questionnaire of the primary endpoint. The data was missing at random. With the imputed data set a multivariate intension to treat analysis was performed. As independent variables age, sex, education, employment status, GAF, social support, the total number of strong and very strong adverse drug effects, the NQ-aspect of social desirability, and the intake of atypical and typical antipsychotics were included in the model. Data processing and statistical calculations were performed with SAS 9.4 (© 2002–2012 by SAS Institute Inc., Cary, North Carolina, USA.).

In a sensitivity analysis the MARS-D score was modelled as a continuous variable. Due to its discrete distribution a Poisson regression was performed in a generalized linear model (GLM). It was necessary to reverse the MARS-D-variable for the Poisson regression because of the left skewed distribution of the data.

## Results

**Fig. 1:**
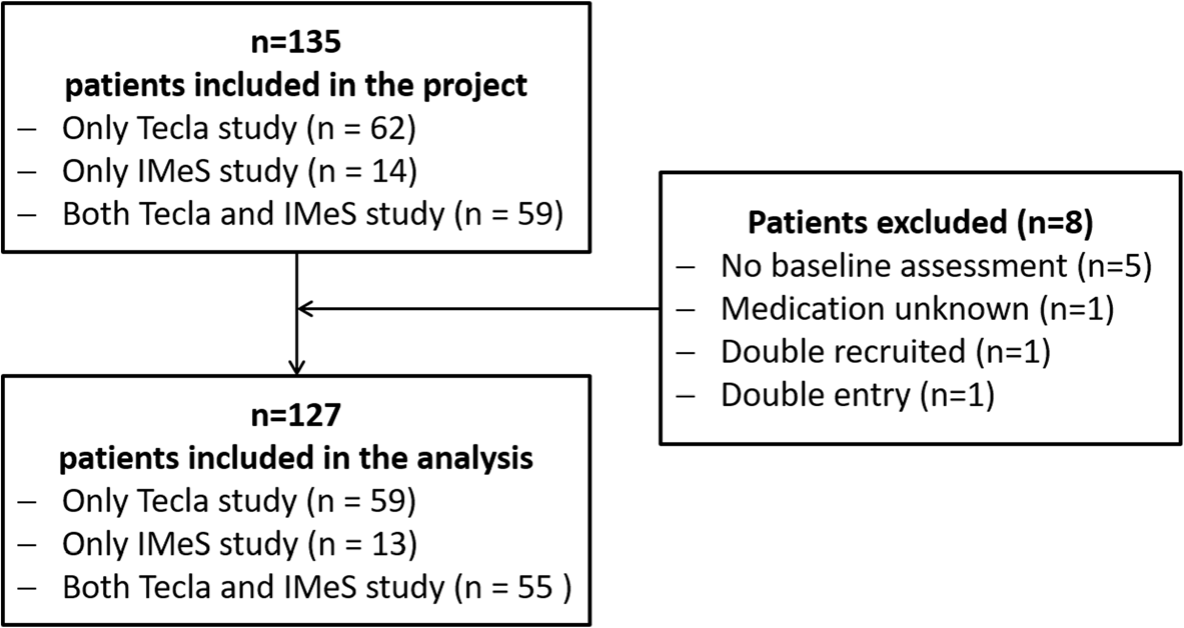
Number of patients included in the analysis

Of 135 participants recruited, 127 could be included in the analyses (Fig. 1).

**Fig. 2:**
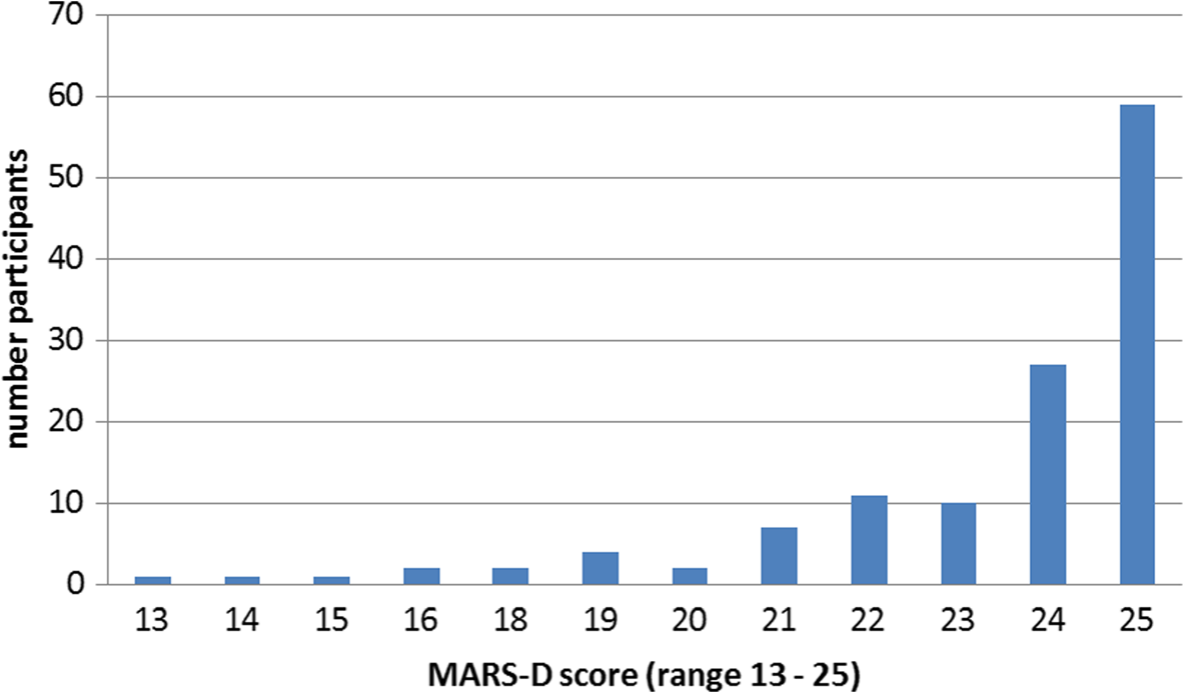
Histogram of the MARS-D score (MARS-D score 25 means complete adherence, < 25 some kind of non-adherence, the lower the MARS-D score the higher is non-adherence)

The participants had a mean age of 42 years (SD 12.9), 57% were men. Eighty-four percent were unemployed and 28% had an education of less than 10 years. One hundred and-six participants had a diagnosis of schizophrenia, schizotypal and delusional disorders (F20 – F29), thereof 72 paranoid schizophrenia (F20), 1 Persistent delusional disorders (F22), 8 acute and transient psychotic disorders (F 23) and 25 schizoaffective disorders (F25). Thirty participants had a diagnosis of mood (affective) disorders (F30 – F39), thereof 27 bipolar affective disorder (F31) and 3 depressive episode (F32). Nine participants had both a diagnosis of schizophrenia, schizotypal and delusional disorders as well as mood (affective) disorders. Atypical antipsychotics were prescribed to 85 participants. Typical antipsychotics were prescribed to 27 participants. Fifteen participants had no prescription for antipsychotics but for drugs of other drug types. Table 1 shows the descriptive results for adherence, global functioning, social support, the number of strong to very strong adverse drug effects, and social desirability. The adherence showed a left skewed distribution (Fig. 2), 54% of the participants reported some kind of non-adherence (MARS-D score < 25).

**Table 1:** Descriptive statistics of the measured variables (Tecla baseline assessment)

	Mean (SD)	Median	Range
Adherence (MARS-D)	23.4 (2.5)	24	13–25
Global functioning (GAF)	54.8 (10.9)	55	30–85
Social support (score)	48.6 (12.9)	51	14–70
Social desirability			
positive qualities (PQ+)	2.7 (0.8)	2.7	0–4
minimize negative qualities (NQ-)	1.1 (0.8)	1	0–4
Number of strong to very strong adverse drug effects	2.6 (1.5)	2	1–10

*SD* standard deviation

**Fig. 3:**
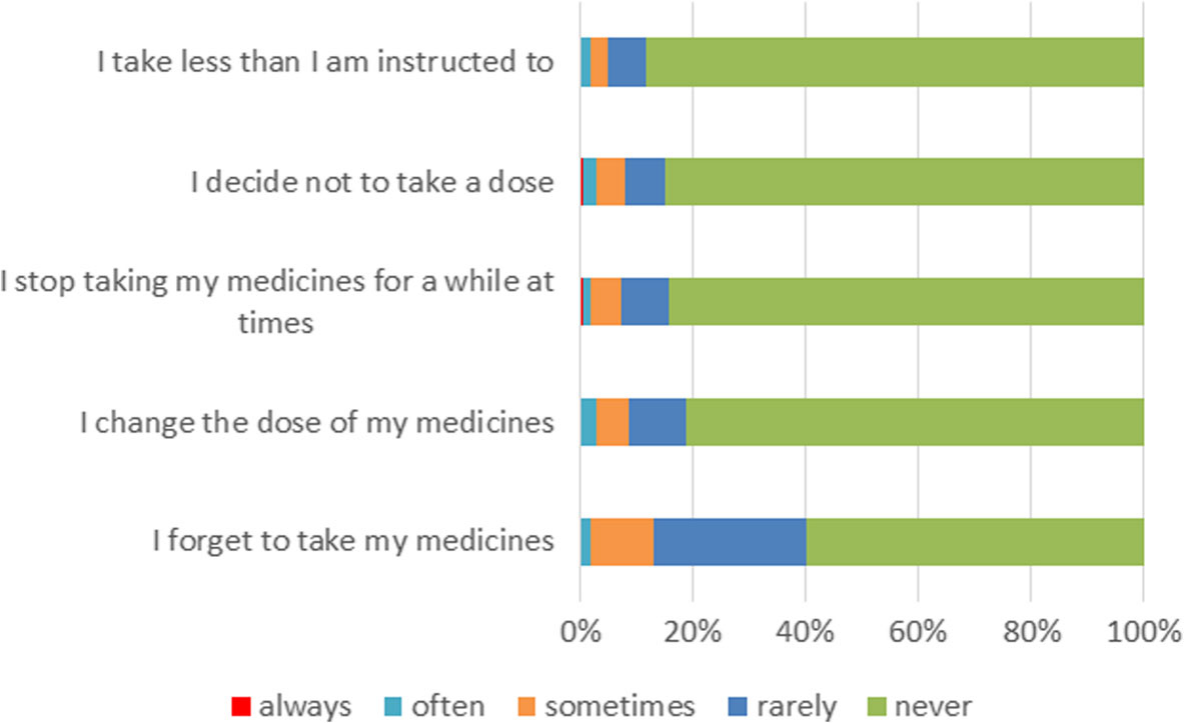
Relative frequencies for reasons of non-adherent behavior assessed with MARS-D

Figure 3 shows the reasons for non-adherence. To forget to take the medicine is the most frequent reason for non-adherent behavior. Active deviation from the prescribed medication scheme (change the dose, stop taking medicines for a while, skip a dose, take less than instructed) were each reported at prevalence of less than 20%.

The results of the logistic regression are shown in Table 2. Higher age, being employed in full time, part time or vocational training, a higher level of global functioning, having more social support and intake of typical antipsychotics have a significant positive influence on adherence. Being female is a negative determinant for adherence. The level of education, the number of strong and very strong adverse drug effects and intake of atypical antipsychotics have no statistically significant effect on adherence.

**Table 2:** Results of the multivariate logistic regression (dependent variable: dichotomized adherence (MARS-D), cut-off score = 24), (being adherent vs. being non-adherent)

	regression coefficient	standard error	*p*-value (α 0.05)	OR	95% CI
Age in years	0.0170	0.0033	< 0.0001	1.017	1.011–1.024
Sex (female vs male)	−0.1557	0.0406	0.0001	0.732	0.625–0.859
Education (≥ 10 years of education vs. < 10 years of education)	0.0026	0.0448	0.9531	1.005	0.843–1.198
Employment status (being employed^a^ vs. not or marginally employed)	0.4507	0.0672	< 0.0001	2.463	1.893–3.206
Global assessment of functioning (GAF)	0.0198	0.0039	< 0.0001	1.02	1.012–1.028
Social desirability (NQ-)	−0.6507	0.0562	< 0.0001	0.522	0.467–0.582
Social support	0.0193	0.0032	< 0.0001	1.02	1.013–1.026
Adverse drug effects	0.0382	0.0255	0.1341	1.039	0.988–1.092
Atypical antipsychotics (atypical drugs vs. other drug types)	−0.1036	0.0627	0.0987	0.813	0.636–1.039
Typical antipsychotics (typical drugs vs. other drug types)	0.4355	0.0728	< 0.0001	2.389	1.796–3.178

*OR* odds Ratio, *CI* confidence interval

^a^ full time, part time, vocational training

A Poisson regression model was performed and used as a sensitivity analysis. The reversal of the MARS-D for the linear Poisson regression also leads to a reversal in the direction of the results. The results (Table 3) are similar to the findings of the primary analysis except for sex and employment status. Patients with lower education, patients that are not or just marginally employed and a lower level of global functioning (GAF) are associated with lesser adherence. Having social support showed no significant impact on medication adherence. The intake of atypical antipsychotics is significantly associated with higher non-adherence whereas the intake of typical antipsychotics is associated with higher adherence.

**Table 3:** Results of the generalized linear Poisson regression. Dependent variable: MARS-D score

	Regression coefficient	Standard error	*p*-value (α 0.05)	beta estimate	95% CI
Age	0,0018	0,0012	0,1321	1,0018	0,9994–1,0042
sex (female vs male)	−0,0873	0,0295	0,0031	0,9164	0,8649–0,9711
Education (≥ 10 years of education vs. < 10 years of education)	−0,2761	0,0321	< 0.0001	0,7587	0,7125–0,8079
Employment status (being employed^a^ vs. not or marginally employed)	−0,2793	0,0523	< 0.0001	0,7563	0,6826–0,8380
Global functioning (GAF)	−0,0246	0,0014	< 0.0001	0,9757	0,9731–0,9784
Social desirability (NQ-)	0,3951	0,0181	< 0.0001	1,4846	1,4329–1,5381
social support	0,0018	0,0012	0,1336	1,0018	0,9995–1,0041
adverse drug effects	0,0152	0,0084	0,0721	1,0153	0,9986–1,0323
Atypical antipsychotics (atypical drugs vs. other drug types)	0,2339	0,0245	< 0.0001	1,2635	1,2043–1,3257
Typical antipsychotics (typical drugs vs. other drug types)	−0,2533	0,0313	< 0.0001	0,7762	0,7300–0,8254

*CI* confidence interval

^a^ full time, part time, vocational training

## Discussion

The MARS-5 was designed to evaluate reasons and prevalences for non-adherent behavior, [23, 40] rather than to measure exact values of the medication use [25, 41, 42]. In the patient group with severe mental disorders, both the primary and the sensitivity analyses showed a positive influence of the global functioning level, of having social support and being employed on adherence.

Medication adherence of patients with severe psychiatric diseases is generally low. The patients in this study were treated in hospitals or day clinics, data assessment was performed shortly before their discharge. However, the proportion of non-adherent patients was relatively high (54%). Stange et al. compared the adherence of patients during the hospital stay with the situation 6 weeks after discharge and found that non-adherence was lower during hospital stay (37.6% vs. 61.2% after 6 weeks) [37]. Hence long-term non-adherence is likely underestimated in our analysis.

Jonsdottir et al. examined medication adherence in patients with severe mental disorders in an ambulant setting [8]. The MARS-5 mean score in this study (22.0) was slightly lower than in our analysis. These authors also found a statistically significant correlation with provider rated medication adherence which supports the validity of the self-rated score used in our study.

In two studies (Mahler et al. [43] and Huther et al. [38]) the adherence of chronically ill patients with MARS-D in primary care settings in Germany was examined. The average MARS-D scores were similar in both studies (mean 23.6 (SD 2.17) [43] and mean 23.5 (SD 2.7) [38]). Mahler et al. reported ‘forget the medication intake’ as the most common cause of non-adherence [43]. These findings correspond with our results. However, Huther et al. found no significant determinants for medication adherence in a subsequent multivariate analysis [38].

Tommelein et al. investigated the accuracy of the MARS-5 for patients with chronic obstructive pulmonary disease (COPD) [40]. The mean adherence for COPD patients was 23.5 (SD = 2.6). 52.9% of patients recorded complete adherence (MARS-5 sum score = 25). Further testing of the MARS-5 showed low sensitivity, specificity, and positive predictive value (PPV). Hence the authors assessed the MARS-5 as inaccurate in identifying non-adherent users of inhalation medication in patients with COPD [40].

Menckeberg et al. used the MARS-5 in a study about inhaled corticosteroids (ICS) in asthma control [41]. In their patient group the mean score value was 19.4 (SD 4.4). Compared to our and others’ findings this score is rather low. However, with 79% scoring above the scale midpoint Menckeberg’s results showed a skewed distribution too. The study showed that many patients were skeptical about the benefits of ICS [41]. This might be one cause for the discrepancy between their results and results of other studies.

In many cases, medication adherence is overestimated based on self-report questionnaires [23, 26–28]. Ose et al. examined the concordance in rating medication adherence among multimorbid patients and their general practitioners (GPs) [44]. Patients often rated their adherence higher than their GPs and only for 20% of the patients medication adherence was rated concordantly. An inherent limitation of self-report questionnaires is that unintentional non-adherence is commonly not assessed [40, 42].

Besides self-reports, adherence can be measured by directly observing the patients while taking their medication, using pill counts, Medication Event Monitoring Systems (MEMS), medical records, medication dispensing records, and pharmacological and biochemical markers [42, 45]. Some of these methods are costly and require increased effort or can only be used for certain drugs. Due to the importance of non-adherent behavior, a simple tool is needed that can easily be implemented in various study settings [8]. Self-reporting questionnaires are more patient-friendly, less expensive and easier to conduct. Jonsdottir et al. validated self-report measures with serum concentrations and found self-report questionnaires a valid method for measuring adherence [8]. The original MARS-5 in English as well as the German version MARS-D are reliable and valid self-report measures of non-adherence [23, 46].

In the literature, predictors for adherence or non-adherence differ. Sendt et al. gives a comprehensive overview [19]. As possible predictors were indicated marriage status, higher education, status of employment, gender, higher subjective well-being, later stage of illness, absence of cannabis use, lower rates of illicit substances and alcohol use, lower rates of medication refusal in early stages of treatment, better therapeutic alliance and higher trust in the physicians [19]. In our findings, higher education showed no significant results but being employed versus not or just marginally being employed showed a strong influence on adherence. Inconsistent predictors were symptom severity, insight, positive attitudes and social support [19]. An Israeli study showed better adherence with having more social support [47]. A majority of studies did not found associations between side effects and adherence [19, 48, 49]. This corresponds to our findings.Our results showed that participants taking typical antipsychotics had a significantly better adherence whereas the intake of atypical psychotics was associated with lower adherence. That result differs from other studies, where adherence was higher in patients taking atypical antipsychotics [14, 49, 50].

In summary, this suggests that adherence apparently is a complex issue [49]. Further research that also considers longitudinal analysis is intended.

## Conclusions

Medication adherence is a complex problem that is influenced by many different parameters [45]. An important finding of this study is that also parameters that are influenceable by interventions like the functioning level or the degree of social support have an effect on adherence. These results can specifically be used for the development of adherence-promoting interventions. For example, knowledge, understanding and support for drug treatment should be strengthened also in the patient’s social environment, among family members and caregivers.

## Abbreviations

CI: Confidence interval
COPD: Chronic obstructive pulmonary disease
eCRF: Electronic Care Report Forms
F-SozU: Social support questionnaire
GAF: Global Assessment Functioning
GP: General practitioner
ICS: inhaled corticosteroids
IMeS: Study “Approaches of individualized medicine in psychiatric disorders”
KSE-G: Short scale Social desirability-gamma questionnaire
MARS: Original Medication Adherence Report Scale in English language
MARS-D: Medication Adherence Report Scale, German version
MEMS: Medication Event Monitoring Systems
NQ-: Minimization of negative qualities (social desirability)
OR: Odds ratio
PPV: positive predictive value
PQ+: Exaggeration of positive qualities (social desirability)
SD: Standard deviation
Tecla: Study “Post stationary **te**lemedi**c**a**l** c**a**re of patients with severe psychiatric disorders”
YLD: years of life lived with disability
